# Tacrolimus as Single‐Agent Immunotherapy for Adult‐Onset Myasthenia Gravis: Remission, Relapse, and Safety

**DOI:** 10.1002/cns.70921

**Published:** 2026-06-01

**Authors:** Zhangyan Geng, Yuting Jiang, Nairong Xie, Wenjia Zhu, Hai Chen, Yaye Wang, Haoran Liu, Qinyao Liu, Congwen Lv, Yu Qian, Yan Lu, Li Di, Min Wang, Min Xu, Xinmei Wen, Bingchuan Xie, Yuwei Da

**Affiliations:** ^1^ Department of Neurology The First Hospital of Hebei Medical University Shijiazhuang Hebei China; ^2^ Department of Neurology Xuanwu Hospital, Capital Medical University Beijing China

**Keywords:** myasthenia gravis, relapse, remission, safety, tacrolimus

## Abstract

**Aims:**

To identify factors influencing remission and relapse, and to evaluate the safety of tacrolimus monotherapy (TAMO) in adult‐onset mild‐to‐moderate myasthenia gravis (MG).

**Methods:**

This retrospective analysis was conducted on MG patients receiving TAMO. Remission was defined as achieving minimal symptom expression (MSE), and multivariable Cox regression analysis identified predictors of remission and relapse. Cancer risk was assessed by standardized incidence ratio (SIR), based on the age‐specific cancer incidence rates in China in 2022.

**Results:**

Among 153 patients, 77.8% achieved MSE, with a median time of 6.0 months. Age at onset (hazard ratio [HR] = 0.982, 95% confidence interval [CI] = 0.968–0.995, *p* = 0.009) and new‐onset MG (HR = 2.065, 95% CI = 1.266–3.368, *p* = 0.004) were independent predictors of time to achieving MSE. The optimal cut‐off value for age at onset was 70 years. Relapse occurred in 31% of patients. Tacrolimus concentration at MSE (HR = 0.815, 95% CI = 0.695–0.956, *p* = 0.012) and dose reduction speed (HR = 1.717, 95% CI = 1.280–2.305, *p* < 0.001) were independent predictors of relapse. The cut‐off values were 5.30 ng/mL and 1.08 mg/year, respectively. Adverse drug reactions (ADRs) occurred in 45.0% of the 200 patients receiving TAMO, with hyperglycemia being the most common (17.5%). Eight patients developed cancer, and the SIR was 2.86 (95% CI = 1.23–5.63, *p* = 0.005) compared with the general Chinese population.

**Conclusion:**

TAMO was associated with higher rates of MSE in adult‐onset mild‐to‐moderate MG patients with new‐onset disease or with an onset age ≤ 70 years. To avoid MG relapse, tacrolimus concentration > 5.30 ng/mL at MSE and dose reduction speed ≤ 1.08 mg/year can be considered. Cancer surveillance may be considered for elderly patients undergoing long‐term tacrolimus therapy. Limitations include the retrospective, uncontrolled design, moderate sample sizes, and limited follow‐up duration; thus, long‐term safety, particularly cancer risk, requires further validation.

## Graphical abstract

Tacrolimus monotherapy yields high remission in adult‐onset MG, especially in new‐onset or younger patients. Relapse is linked to low tacrolimus concentration and rapid tapering. Hyperglycemia was the most common ADR. Long‐term use may increase cancer risk.

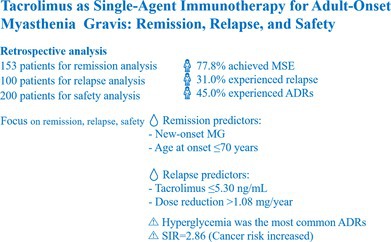

## 1 Introduction

Myasthenia gravis (MG) is an acquired autoimmune disorder affecting the neuromuscular junction, primarily mediated by autoantibodies targeting the postsynaptic membrane, leading to fluctuating muscle weakness and fatigability [1]. Most MG patients require long‐term immunosuppressive therapy, with glucocorticoids generally serving as the first‐line treatment [2]. However, long‐term glucocorticoid therapy is often limited by severe adverse events. The exacerbation of comorbidities such as severe osteoporosis, poorly controlled diabetes or hypertension is a major concern, particularly in the elderly population [3]. Furthermore, an appreciable number of patients refuse glucocorticoids due to concerns about side effects [4, 5]. To minimize the risk of disease relapse during tapering and to limit the side effects of long‐term steroid treatment, many immunosuppressant medications are used as steroid‐sparing agents [6]. Tacrolimus, one of these agents, is a macrolide molecule that can inhibit the proliferation of activated T‐cells [7]. It has a faster onset of action than azathioprine (AZA) or mycophenolate mofetil (MMF) [8, 9, 10] and is less nephrotoxic than cyclosporine A (CsA) [11]. These advantages have led to the widespread adoption of tacrolimus as a steroid‐sparing agent in the management of MG. In such clinical scenarios—where glucocorticoids are contraindicated or refused—tacrolimus monotherapy (TAMO) is considered as an alternative primary strategy, particularly for patients with mild‐to‐moderate MG^5^.

Several studies have demonstrated the efficacy of TAMO as a single‐agent immunotherapy in patients with MG [4, 5, 12, 13]. However, these studies have reported inconsistent findings regarding factors influencing its therapeutic efficacy [5, 12, 13]. The limitations of these studies include small sample sizes, short follow‐up periods, and a focus on specific subtypes of MG, such as ocular MG, or the inclusion of patients with varying ages of onset, including juvenile‐onset MG. Data on the incidence of relapse with TAMO remain limited. In our previous study, we analyzed 57 MG patients treated with TAMO as their initial immunotherapy regimen, and no factors affecting relapse were found [5]. In contrast, two studies have specifically investigated relapse associated with tacrolimus dose reduction, both of which reported an association between the rate of dose reduction and relapse risk. However, these studies focused on tacrolimus in combination with prednisone rather than as monotherapy [14, 15]. Furthermore, the long‐term safety profile of TAMO has not yet been comprehensively evaluated in a large group of MG patients.

In this study, we investigate the factors influencing time to remission and relapse in adult‐onset mild‐to‐moderate MG patients receiving TAMO as a single‐agent immunotherapy. Furthermore, we conduct a comprehensive analysis of the adverse effects associated with TAMO in MG patients.

## 2 Methods

### 2.1 Participants and Study Design

We conducted a retrospective analysis of participants registered in the Myasthenia Gravis Trial Database at Xuanwu Hospital, Capital Medical University, from February 2017 to November 2024. This study was approved by the Ethics Committee of Xuanwu Hospital (No. 2017084), and each participant provided written informed consent. Given the retrospective, single‐center, and uncontrolled design of this study, the observed rates of remission, relapse, and adverse events represent real‐world observational data from a specifically selected cohort and should be interpreted as descriptive absolute outcomes rather than comparative efficacy.

MG was diagnosed based on fluctuating muscle weakness and at least one positive result of the following tests: pharmacological, serological, and electrophysiologic tests [8]. Of the 1738 MG patients in the database, 712 patients who received tacrolimus therapy were screened. The exclusion criteria were as follows: (a) receipt of intravenous immunoglobulin (IVIG), plasma exchange (PE), or glucocorticoids within 1 month, or other immunosuppressive (IS) drugs within 3 months prior to tacrolimus initiation; (b) insufficient baseline data or lost to follow‐up; (c) patients with Myasthenia Gravis Foundation of America (MGFA) class IV or V; or (d) onset age < 18 years. A total of 200 eligible patients who received TAMO were included. The baseline was defined as the time of tacrolimus initiation. Concurrent use of cholinesterase inhibitor was permitted.

For the remission analysis, additional exclusion criteria were applied to eligible patients based on: (a) thymectomy within 6 months before tacrolimus treatment; (b) a baseline Myasthenia Gravis Activities of Daily Living (MG‐ADL) score ≤ 1, defined as ‘minimal symptom expression’ (MSE) [16]; or (c) tacrolimus treatment < 1 month. Remission was defined as achieving MSE, and patients were categorized into the MSE group and non‐MSE group.

For the relapse analysis, we followed up patients who achieved MSE. Relapse of MG was defined as clinical worsening—indicated by an increase in the MG‐ADL score of ≥ 2 points [17]. Patients were excluded if they relapsed with definite precipitating factors, such as infection, inappropriate drug use, fatigue, surgery, or psychological factors (e.g., anxiety). The remaining patients were categorized into relapsed group and non‐relapsed group. Relapse time was defined as the interval from MSE achievement to the occurrence of relapse.

**FIGURE 1:**
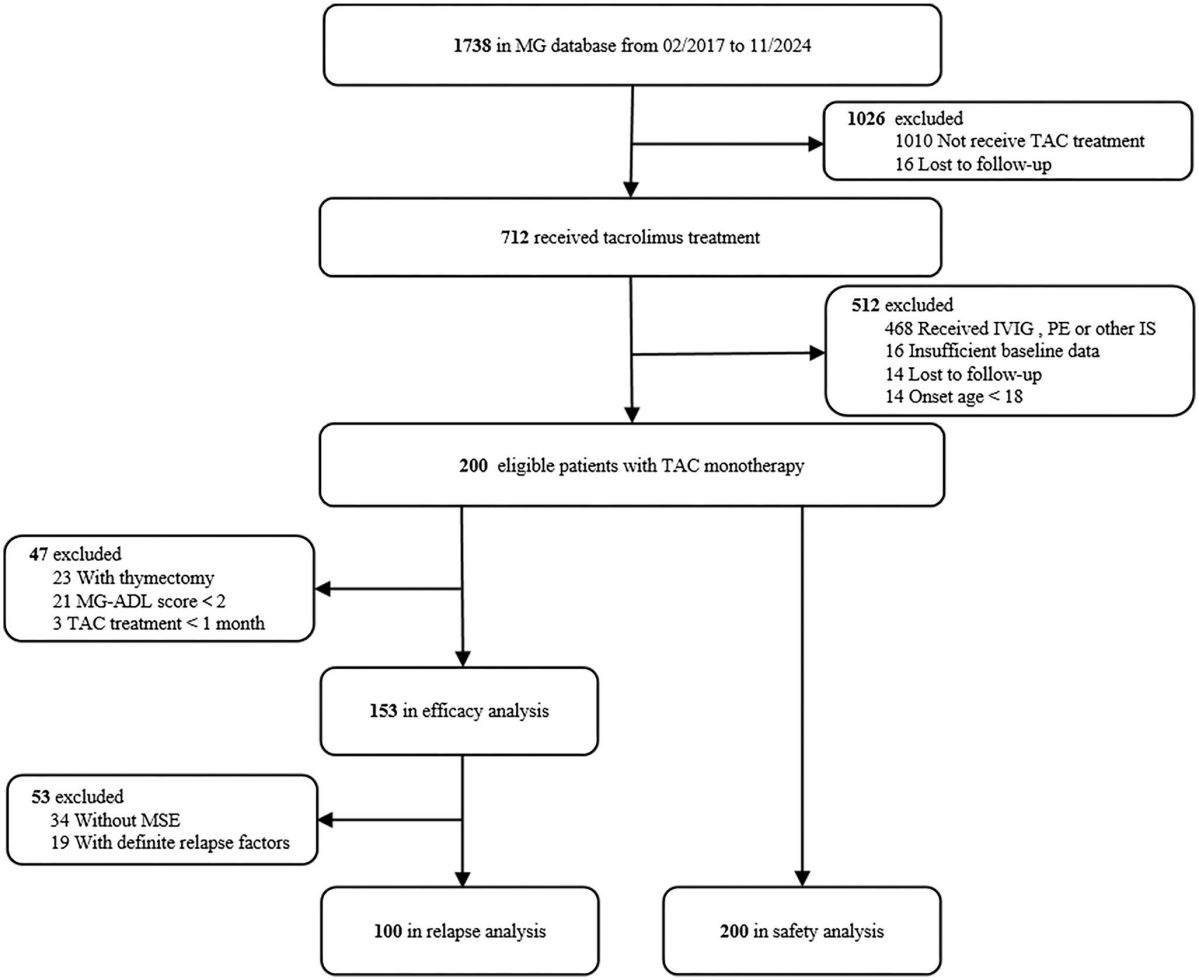
Flowchart of the participants included in the current study. IS, Immunosuppressive; IVIG, intravenous immunoglobulin; MG, myasthenia gravis; MG‐ADL, Myasthenia Gravis Activities of Daily Living; MSE, minimal symptom expression; PE, plasma exchange; TAC, tacrolimus.

For the safety analysis, all eligible patients with TAMO were included. Adverse drug reactions (ADRs) were used to assess the safety of TAMO. ADRs were defined as any adverse events considered to be at least possibly related to TAMO based on the assessment of the treating physician or for which assessment of causality was missing [18]. The selection procedure is depicted in Figure 1.

### 2.2 Data Collection and Tacrolimus Therapeutic Regimens

Demographic and clinical data at baseline were collected from the MG database, including sex, age at onset, age at tacrolimus initiation, MGFA classification, MG‐ADL score, quantitative myasthenia gravis (QMG) score, thymoma, thymectomy, repetitive nerve stimulation (RNS), antibody status, disease duration, and comorbidities. New‐onset MG was defined as the onset of symptoms within 12 months prior to tacrolimus initiation [19]. MG‐ADL scores, tacrolimus dosage, and ADRs associated with TAMO were assessed during follow‐up visits at 1, 3, 6, 9, and 12 months, with subsequent assessments every 6 months. Key ADRs included hyperglycemia, renal impairment, joint pain, and cancers. Prior to initiating tacrolimus, patients were screened to exclude the cancers.

The management of myasthenia gravis at our center is guided by the International Consensus Guidance for Management of Myasthenia Gravis (2016) [20]. TAMO was primarily prescribed to mild‐to‐moderate MG patients with specific contraindications to glucocorticoids (e.g., severe osteoporosis, poorly controlled diabetes or hypertension), or for those who explicitly refused such therapies due to safety concerns. All patients were initially prescribed tacrolimus at a daily dose of 2 mg, with the aim of achieving a target trough concentration of 4.8–10 ng/mL [21]. The dosage of tacrolimus was adjusted based on clinical efficacy, side effects, and trough concentration. Tapering of tacrolimus was considered if remission had been sustained for at least 6 months. The dosage was reduced by no more than 1 mg per day every 6 months [5]. In the event of disease fluctuation during dose reduction, the tacrolimus dose was increased back to the maintenance level, or the patient was switched to an alternative immunosuppressant. The final dose was defined as the dose at the last follow‐up if no relapse occurred, the dose at relapse if it occurred, or 0 if tacrolimus was withdrawn. The taper duration was defined as the time interval from the initiation of the maximum dose before MSE to the start of the final dose. Dose reduction speed was calculated as the difference between the maximum dose and the final dose, divided by the taper duration.

### 2.3 Statistical Analysis

Continuous variables with a non‐normal distribution were presented as median with interquartile range [IQR], while categorical variables were presented as counts and percentages. Group comparisons were performed using the Mann–Whitney U Test for continuous variables and chi‐square test or Fisher's exact test for categorical variables. Kaplan–Meier (K‐M) analysis was conducted to estimate the cumulative probability of achieving MSE. The Cox regression model was used to investigate the association between event occurrences (MSE achievement or relapse) and multiple variables. Patients who had not experienced these events were right‐censored at their last follow‐up. Variables with *p* < 0.2 in univariate analysis were included in the multivariate analysis. The X‐tile software (Version 3.6.1, Yale University School of Medicine) was used to identify the optimal cut‐off values for age at onset, tacrolimus concentration at MSE, and dose reduction speed. To evaluate the cancer risk related to tacrolimus therapy, the standardized incidence ratio (SIR) with its 95% confidence interval (CI) and *p*‐value were calculated, compared with the general Chinese population. The expected number of cancer cases was estimated based on the age‐specific incidence rates in China in 2022, as reported by the National Central Cancer Registry of China [22]. All data were analyzed using SPSS (Version 25, IBM, Armonk, NY, USA) and R (Version 4.4.0,R Foundation for Statistical Computing, Vienna, Austria). A two‐tailed *p* < 0.05 was considered statistically significant.

## 3 Results

### 3.1 Remission Analysis of TAMO for Achieving MSE

A total of 153 adult‐onset mild‐to‐moderate MG patients treated with TAMO were included in the remission analysis. Females accounted for 55.6%. The median age at onset was 61.0 years (IQR 49.5–68.0), with 75.2% (115/153) patients having disease onset at 50 years or older, and 38.6% (59/153) at 65 years or older. The median follow‐up time after tacrolimus initiation was 24.4 months (IQR 10.6–43.4). At baseline, MG‐ADL and QMG scores were 5 (IQR 3–6) and 7 (IQR 5–11), respectively. According to MGFA classification, 22.2% (34/153) of patients presented with MGFA class I. In terms of diagnostic tests, 87.9% (131/149) were anti‐AChR‐positive and 62.3% (71/114) were RNS positive. Thymoma was present in 9.8% (14/153) of patients. The median disease duration was 7.0 months (IQR 2.3–21.8), and 60.1% (92/153) patients had new‐onset MG (Table S1).

In remission analysis, 77.8% (119/153) patients achieved MSE. The cumulative probability of achieving MSE was 73.5%, 86.0%, and 94.5% at 12, 24, and 36 months, respectively. The estimated median period to MSE was 6.0 months (95% CI = 5.3–6.7) (Figure S1). It is important to note that due to the retrospective and uncontrolled design, these observed rates may be subject to selection bias and should be interpreted as descriptive outcomes rather than definitive comparative efficacy.

The baseline MG‐ADL score was significantly lower in the MSE group compared to the non‐MSE group (5 vs. 6, *p* = 0.035). However, there were no significant differences between the two groups in other characteristics, including sex, age at onset, MGFA classification, thymoma, thymectomy, RNS, antibody status, tacrolimus concentrations at 1 month, disease duration, new‐onset MG, and comorbidities (Table 1). Multivariable Cox regression analysis revealed that age at onset (hazard ratio [HR] = 0.982, 95% CI = 0.968–0.995, *p* = 0.009) and new‐onset MG (HR = 2.065, 95% CI = 1.266–3.368, *p* = 0.004) were independent predictors of the time to achieving MSE with TAMO (Table 2). Furthermore, using X‐tile software, we determined the cut‐off value for age at onset to be 70 years. Patients with an onset age of ≤ 70 years were more likely to achieve MSE (Figure S2).

**TABLE 1:** The comparison of clinical features between MSE and non‐MSE groups.

	MSE (*n* = 119)	Non‐MSE (*n* = 34)	*p*
Female, *n* (%)	65 (54.6)	20 (58.8)	0.664
Age at onset, years (IQR)	61.0 (50.0–68.0)	58.5 (48.5–70.0)	0.823
MGFA class I at baseline, *n* (%)	29 (24.4)	5 (14.7)	0.232
MG‐ADL score at baseline, (IQR)	5 (3–6)	6 (4–7)	**0.035**
QMG score at baseline, (IQR)	7 (5–10)	7 (5–12)	0.442
Thymoma, *n* (%)	10 (9.0)	4 (13.3)	0.720
Thymectomy, *n* (%)	11 (9.2)	4 (12.1)	0.872
RNS (+), *n* (%)	53 (60.2)	18 (69.2)	0.405
AChR‐Ab (+), *n* (%)	104 (88.1)	27 (87.1)	> 0.999
Adequate TAC concentrations (4.8–10 ng/mL) at 1 month, *n* (%)	56 (56.6)	12 (57.1)	0.961
Disease duration, months (IQR)	6.0 (2.2–18.2)	14.7 (2.9–37.9)	0.137
New‐onset MG, *n* (%)	75 (63.0)	17 (50.0)	0.171
Comorbidities, *n* (%)			
Hypertension	50 (42.0)	13 (38.2)	0.693
Diabetes	25 (21.0)	5 (14.7)	0.414
Coronary heart disease	16 (13.4)	2 (5.9)	0.365
Thyroid disease	11 (9.2)	3 (8.8)	> 0.999
Autoimmune disease	5 (4.2)	1 (2.9)	> 0.999

*Note:* Bold values in table indicate *p* < 0.05.

Abbreviations: AChR‐Ab, acetylcholine receptor antibodies; IQR, interquartile range; MG, myasthenia gravis; MG‐ADL, Myasthenia Gravis Activities of Daily Living; MGFA, Myasthenia Gravis Foundation of America classification; MSE, minimal symptom expression; QMG, quantitative myasthenia gravis; RNS, repetitive nerve stimulation; TAC, tacrolimus.

**TABLE 2:** Variables associated with time to MSE predicted by Cox regression model.

Variables	Univariable		Multivariable	
	HR(95% CI)	*p*	HR(95% CI)	*p*
Female	0.733 (0.509–1.056)	**0.095**	0.810 (0.526–1.248)	0.339
Age at onset	0.988 (0.977–1.000)	**0.045**	0.982 (0.968–0.995)	**0.009**
MGFA class I at baseline	1.176 (0.773–1.791)	0.449		
MG‐ADL score at baseline	0.900 (0.831–0.976)	**0.010**	0.927 (0.838–1.025)	0.141
QMG score at baseline	0.976 (0.934–1.020)	0.283		
Thymectomy	0.657 (0.353–1.224)	**0.186**	0.717 (0.339–1.520)	0.386
AChR‐Ab (+)	0.712 (0.405–1.251)	0.237		
RNS (+)	0.729 (0.472–1.124)	**0.152**	0.959 (0.605–1.519)	0.858
New‐onset MG	1.815 (1.244–2.650)	**0.002**	2.065 (1.266–3.368)	**0.004**
Comorbiditie				
Hypertension	1.197 (0.829–1.727)	0.338		
Diabetes	1.115 (0.717–1.726)	0.628		
Coronary heart disease	1.027 (0.606–1.742)	0.921		
Thyroid disease	0.622 (0.331–1.169)	**0.140**	0.824 (0.406–1.672)	0.591
Autoimmune disease	1.233 (0.500–3.042)	0.649		

*Note:* Variables were included in multivariable analysis if *p* < 0.20 in univariable analyses. Bold values in table: *p* < 0.2 (univariable), *p* < 0.05 (multivariable).

Abbreviations: AChR‐Ab, acetylcholine receptor antibodies; HR, hazard ratio; MG, myasthenia gravis; MG‐ADL, Myasthenia Gravis Activities of Daily Living; MGFA, Myasthenia Gravis Foundation of America classification; MSE, minimal symptom expression; QMG, quantitative myasthenia gravis; RNS, repetitive nerve stimulation.

### 3.2 Predictors of Relapse in Tacrolimus‐Induced Remission

With a median follow‐up of 26.1 months (IQR 10.6–40.7) after remission, 100 patients were included in relapse analysis, with 31.0% (31/100) patients experienced relapse. The median relapse time in relapsed group was 13.8 months (IQR 6.5–25.7). Compared with the non‐relapsed group, the relapsed group had a significantly higher proportion of patients with tacrolimus dose reduction (96.8% vs. 59.4%, *p* < 0.001), a faster dose reduction speed (1.01 mg/year vs. 0.58 mg/year, *p* < 0.001), lower tacrolimus concentrations at MSE (5.3 ng/mL vs. 7.0 ng/mL, *p* = 0.028), and longer follow‐up time (33.6 months vs. 19.3 months, *p* = 0.004) (Table 3). Multivariable Cox regression analysis identified that tacrolimus concentration at MSE (HR = 0.815, 95% CI = 0.695–0.956, *p* = 0.012) and dose reduction speed (HR = 1.717, 95% CI = 1.280–2.305, *p* < 0.001) were independent predictors of time to relapse in tacrolimus‐induced remission (Table 4). X‐tile software identified the cut‐off values of tacrolimus concentration at MSE and dose reduction speed as 5.30 ng/mL and 1.08 mg/year, respectively (Figure S3A,B).

**TABLE 3:** Comparison of clinical characteristics between the relapsed group and non‐relapsed group.

	Total (*n* = 100)	Non‐relapsed (*n* = 69)	Relapsed (*n* = 31)	*p*
Female, *n* (%)	51 (51.0)	37 (53.6)	14 (45.2)	0.434
Age at onset, years (IQR)	61.0 (50.0–68.0)	58.0 (40.75–68.0)	65.0 (57.5–68.0)	0.114
MGFA class I at baseline, *n* (%)	25 (25.0)	20 (29.0)	5 (16.1)	0.170
MG‐ADL score at baseline, (IQR)	5 (3–6)	4 (3–6)	5 (4–6)	0.720
QMG score at baseline, (IQR)	7 (5–10)	7 (5–10)	8 (6–10)	0.773
Thymoma, *n* (%)	9 (9.0)	7 (10.6)	2 (7.1)	0.890
Thymectomy, *n* (%)	8 (8.0)	7 (10.1)	1 (3.2)	0.435
AChR‐Ab (+), *n* (%)	85/99 (85.9)	61 (88.4)	24 (80.0)	0.270
RNS (+), *n* (%)	43/74 (58.1)	31 (57.4)	12 (60.0)	0.841
Disease duration, months (IQR)	5.7 (2.6–18.1)	5.6 (2.6–18.4)	6.0 (2.7–18.1)	> 0.999
Time to attain MSE, months (IQR)	4.6 (2.7–7.8)	5.0 (2.8–7.6)	3.7 (2.7–8.6)	0.671
TAC dose reduction, *n* (%)	71 (71.0)	41 (59.4)	30 (96.8)	**< 0.001**
TAC dose reduction speed, mg/year (IQR)	0.73 (0–1.36)	0.58 (0–1.04)	1.01 (0.56–1.60)	**< 0.001**
TAC concentrations at MSE, ng/mL (IQR)	6.7 (4.7–8.2)	7.0 (5.6–9.7)	5.3 (4.0–7.2)	**0.028**
Follow‐up time, months (IQR)	26.1 (10.6–40.7)	19.3 (8.6–37.8)	33.6 (19.7–44.4)	**0.004**

*Note:* nerve stimulation. Bold values in table indicate *p* < 0.05.

Abbreviations: AChR‐Ab, acetylcholine receptor antibodies; IQR, interquartile range; MG‐ADL, Myasthenia gravis activities of daily living; MGFA, Myasthenia gravis foundation of America classification; MSE, minimal symptom expression; QMG, quantitative myasthenia gravis; RNS, repetitive; TAC, tacrolimus.

**TABLE 4:** Variables associated with time to relapse predicted by Cox regression model.

Variables	Univariable		Multivariable	
	HR (95% CI)	*p*	HR (95% CI)	*p*
Female	0.833 (0.406–1.708)	0.618		
Age at onset	1.024 (0.992–1.057)	**0.142**	1.047 (0.995–1.102)	0.075
MGFA class I at baseline	0.450 (0.172–1.178)	**0.104**	0.425 (0.136–1.331)	0.142
MG‐ADL score at baseline	1.062 (0.903–1.250)	0.467		
QMG score at baseline	1.037 (0.946–1.136)	0.439		
Thymectomy	0.486 (0.066–3.576)	0.479		
AChR‐Ab (+)	0.667 (0.268–1.660)	0.384		
RNS (+)	1.622 (0.660–3.985)	0.292		
Time to attain MSE	1.023 (0.982–1.067)	0.276		
TAC dose reduction speed	1.426 (1.191–1.707)	**< 0.001**	1.717 (1.280–2.305)	**< 0.001**
TAC concentrations at MSE	0.847 (0.721–0.995)	**0.044**	0.815 (0.695–0.956)	**0.012**

*Note:* Variables were included in multivariable analysis if *p* < 0.20 in univariable analyses. Bold values in table: *p* < 0.2 (univariable), *p* < 0.05 (multivariable).

Abbreviations: AChR‐Ab, acetylcholine receptor antibodies; HR, hazard ratio; MG‐ADL, Myasthenia Gravis Activities of Daily Living; MGFA, Myasthenia Gravis Foundation of America classification; MSE, minimal symptom expression; QMG, quantitative myasthenia gravis; RNS, repetitive nerve stimulation; TAC, tacrolimus.

We continued follow‐up monitoring for relapsed patients. Among them, none required hospitalization, two were switched to other immunosuppressants, and the remaining patients showed clinical improvement after an increased tacrolimus dosage.

### 3.3 Safety Analysis of TAMO

A total of 200 patients with TAMO were included in the safety analysis. Females accounted for 50.0%, and the median age at tacrolimus initiation was 61.0 years (IQR 46.25–68.0). At baseline, 88.2% (164/186) of patients were anti‐AChR‐positive, 64.5% (91/141) were RNS positive, and thymoma was identified in 12.7% (24/189) of patients. The median disease duration was 7.1 months (IQR 2.5–23.0). Regarding comorbidities, hypertension was present in 40.5% of patients, diabetes mellitus in 19.5%, and coronary atherosclerotic heart disease in 10.5% (Table S2).

With a median follow‐up of 22.3 months (IQR 7.5–40.5), 127 ADRs were reported by 45.0% (90/200) patients. Specially, the most frequently reported ADRs (occurring in ≥ 5% of patients) were hyperglycemia (17.5%), gastrointestinal symptoms (8.0%), renal impairment (7.0%), tremor (6.5%), and hyperuricemia (6.0%). Sixteen patients (8%) withdrew tacrolimus treatment due to adverse drug reactions (ADRs) at a median time of 5.5 months (IQR 2.5–13.9)(Table 5). All ADRs, except for cancers, resolved following dose reduction or drug withdrawal.

**TABLE 5:** Incidence and occurring time of ADRs reported by patients.

	*n* (%)	Time, months, (IQR)
Patients with ADRs	90 (45.0)	5.0 (1.6–17.0)
**Discontinuation** due to ADRs	16 (8.0)	5.5 (2.5–13.9)
ADRs		
Hyperglycemia	35 (17.5)	2.3 (0.9–16.3)
Gastrointestinal symptoms	16 (8.0)	2.8 (1.1–4.8)
Renal impairment	14 (7.0)	5.6 (3.2–11.0)
Tremor	13 (6.5)	6.3 (2.8–26.1)
Hyperuricemia	12 (6.0)	4.2 (1.9–10.7)
Joint pain	11 (5.5)	5.3 (2.0–31.1)
High blood pressure	5 (2.5)	3.0 (0.7–29.7)
Palpitation	4 (2.0)	2.2 (0.6–15.7)
Increased liver enzymes	3 (1.5)	1.9 (1.7–4.5)
Leg cramps	2 (1.0)	7.2 (0.6–13.7)
Infection	2 (1.0)	12.6 (11.5–13.7)
Blood cell count abnormalities	2 (1.0)	7.2 (0.3–14.0)
Electrolyte Imbalance	1 (0.5)	1.0 (1.0)
Menstrual disorder	1 (0.5)	3.0 (3.0)
Cancer	8 (4.0)	35.5 (28.9–46.3)
Lung cancer	3 (1.5)	37.5 (35.5–38.6)
Cardia carcinoma	1 (0.5)	30.3 (30.3)
Gallbladder cancer	1 (0.5)	20.2 (20.2)
Breast cancer	1 (0.5)	28.4 (28.4)
Myeloma	1 (0.5)	48.4 (48.4)
Renal cancer	1 (0.5)	52.0 (52.0)

Abbreviations: ADRs, adverse drug reactions; IQR, interquartile range.

Hyperglycemia developed in 17.5% (35/200) of patients at a median of 2.3 months (IQR 0.9–16.3) following tacrolimus initiation. These patients were generally older, with a median age of 64 years. Of the 35 affected patients, 12 had pre‐existing diabetes mellitus. In these 12 patients, glycemic control was managed by lifestyle modifications alone in 1 patient, tacrolimus dose reduction alone in 4, adjustment of hypoglycemic agents alone in 5, and a combination of tacrolimus dose reduction and hypoglycemic agent adjustment in 2. Among the 23 patients with new‐onset hyperglycemia, management strategies were as follows: lifestyle interventions alone (*n* = 16), tacrolimus dose reduction alone (*n* = 2), switching to alternative immunosuppressants (*n* = 2), initiation of hypoglycemic agents alone (*n* = 1), and a combination of tacrolimus dose reduction with initiation of hypoglycemic agents (*n* = 2).

Eight (4%) patients developed various types of cancer, and four of these patients died from cancer (Table 5). In detail, the malignancies observed were three cases of lung cancer and one case each of cardia carcinoma, breast cancer, gallbladder cancer, renal cancer, and myeloma. The median time from tacrolimus initiation to cancer diagnosis was 35.5 months (IQR 28.9–46.3). All eight patients were over the age of 60 years. Furthermore, we evaluated the cancer risk related to tacrolimus in MG patients, compared with the general population. The expected number of cancer cases was 2.80, and the SIR of cancer was 2.86 (95% CI = 1.23–5.63, *p* = 0.005).

## 4 Discussion

We observed that TAMO was associated with the achievement of MSE in adult‐onset mild‐to‐moderate MG patients, particularly in those with new‐onset MG and those aged ≤ 70 at onset. Tacrolimus concentration at MSE ≤ 5.30 ng/mL and dose reduction speed > 1.08 mg/year were independent predictors of relapse. It is worth noting that caution was warranted regarding the risk of cancers associated with TAMO.

In our study, the estimated median time to achieve MSE was 6.0 months, with a cumulative probability of 73.5% at 12 months, indicating the potential clinical benefit of TAMO. This finding is consistent with previous studies that reported remission rates ranging from 69.2% to 73.5% after 12 months of TAMO [4, 13]. We identified specific factors influencing the time to achieve MSE, noting that patients with new‐onset MG and those aged ≤ 70 at onset were more likely to benefit from TAMO. The advantages of early immunotherapy intervention have been well‐documented and are supported by Japanese clinical guidelines [5, 23, 24, 25]. Long‐term structural changes at the neuromuscular junction, characterized by loss of synaptic folds, widening of the clefts, and nerve terminal relocation, may hinder therapeutic efficacy, highlighting the importance of early immunotherapy [24].

Age emerged as a significant factor in response to TAMO. Duan et al. [13] reported that MG patients aged < 39 exhibited a better response to TAMO; however, their study included participants of various ages, including those with juvenile‐onset MG. In contrast, our research specifically focused on adult‐onset mild‐to‐moderate MG patients. In our investigation, patients receiving TAMO tended to be older, possibly due to the apprehension of elderly patients regarding corticosteroid use [5]. In another study, among MG patients aged ≥ 65 years, only 43% achieved remission with tacrolimus treatment [26]. Notably, 43.8% of patients in our study who discontinued tacrolimus due to ADRs were aged 65 or older. We hypothesize that reduced medication efficacy in elderly MG patients may result from the impact of ADRs. Moreover, inadequate responses to tacrolimus in the elderly could be attributed to immunosenescence and inflammaging, which may diminish both innate and adaptive immunity, thus impairing the ability to mount an adequate immune response [25, 27]. Additionally, Duan et al. and Itani et al. suggested TAMO for patients with lower QMG or MG‐ADL scores [12, 13]. We found that the MG‐ADL score was significantly lower in the MSE group, indicating that patients with severe MG may be less likely to achieve MSE with TAMO. Uzawa et al. found that early fast‐acting treatment (EFT) was efficacious for all severities of MG^26^. For patients with severe MG, we recommend initiating EFT or other biological therapies with rapid onset of action.

We observed a relapse rate of 31.0% among patients receiving TAMO for MG. Previous investigations, not limited to tacrolimus therapy, have reported varying relapse rates. A comprehensive study conducted across seven major neurological centers in China documented a relapse rate of 24.1% [28]. Conversely, Nishida et al. reported that 4% of patients with well‐controlled MG experienced an exacerbation following tacrolimus tapering [15]. These differences in relapse rates likely reflect the varying definitions of relapse used across studies. We defined relapse strictly as clinical worsening (an MG‐ADL increase of ≥ 2 points). In contrast, Nishida et al. defined exacerbation as occurring within 3 months after dose reduction, which appears to capture primarily early and rapid recurrences. The definition used in our study may offer a broader and more inclusive measure of long‐term disease instability under TAMO. We found only one patient relapsed without dose reduction. Regarding clinical management, our data showed that most relapsed patients regained clinical stability after restoring the tacrolimus dosage to maintenance levels. For patients who fail to achieve MSE or experience severe relapses, our clinical pathway typically involves transitioning to alternative therapies. Depending on patient tolerance and disease severity, this include the addition of low‐dose glucocorticoids, switching to other traditional immunosuppressants, or escalating to biologic agents such as efgartigimod, rituximab, or telitacicept. We identified the concentration of tacrolimus at MSE and dose reduction speed as critical factors associated with relapse of MG. It is well established that maintaining an adequate trough serum concentration of tacrolimus (4.8–10 ng/mL) is associated with a higher likelihood of achieving remission [21]. Additionally, our findings indicated that patients with elevated serum concentrations of tacrolimus did not experience relapses. To avoid disease rebound, the concentration of tacrolimus should be more than 5.30 ng/mL. Furthermore, we suggest that tapering the dosage by more than 1.08 mg/year should be avoided. Nishida et al. [15] reported that the reduction of tacrolimus more than 1.5 mg/day was related to exacerbation in patients with well‐controlled anti‐AChRAb‐positive MG, while they did not analyze the duration of tapering. Another investigation found that to prevent MG relapse, the reduction of tacrolimus should not exceed 0.76 mg/year [14]. Both of these studies employed a therapeutic approach combining tacrolimus with prednisone and focused on relapses occurring within 3 to 6 months following tacrolimus tapering [14, 15]. In contrast, our research specifically examined relapses during TAMO.

In our study, 45.0% of MG patients receiving TAMO reported ADRs, primarily in the early months of treatment. Hyperglycemia, a well‐recognized tacrolimus ADR, occurred in 17.5% of our study, exceeding rates from prior studies [4, 5, 13], potentially due to their limited sample sizes or younger participant demographics. Patients who developed hyperglycemia in this study were older, with a median age of 64 years. Thus, elderly MG patients need more frequent monitoring of plasma glucose when taking tacrolimus. Among patients with hyperglycemia, the majority of those with pre‐existing diabetes mellitus required tacrolimus dose reduction and/or adjustment of hypoglycemic agents, while a proportion of those with new‐onset hyperglycemia could manage their blood glucose through lifestyle interventions alone. Additionally, tacrolimus is commonly combined with prednisone for MG treatment, and both drugs influence glycemic metabolism. Further research is needed to elucidate the impact of both on glycemic regulation to optimize clinical management. Striking a balance between effective symptom control and minimizing ADRs remains a significant challenge in the treatment of MG.

In this study, 4.0% of patients developed malignancies. Given that the median time to cancer diagnosis was nearly three years after treatment initiation, the relatively short follow‐up duration may lead to an underestimation of the cancer risk. An elevated SIR was observed in MG patients on tacrolimus, indicating a significantly higher cancer incidence compared to the general population. This finding aligns with a study in Sweden that reported an association between corticosteroid‐sparing immunosuppressants and increased cancer risk [29]. While most previous studies in MG patients with tacrolimus did not report malignancies [4, 5, 12, 13, 26, 30, 31]. Tacrolimus‐related cancer risk varies across diseases, with reported incidences of 2% in inflammatory bowel disease, 2.4% in lupus nephritis, and 19% post‐liver transplantation [18, 32, 33]. However, not all studies report a heightened cancer risk with tacrolimus. For instance, one study in patients with systemic lupus erythematosus (SLE) found that calcineurin inhibitor use was not associated with a significant increase in cancer risk, with a SIR of 1.08 [34]. Similarly, kidney transplant recipients on tacrolimus/mycophenolate mofetil showed no elevated risk for colorectal cancer (SIR = 0.79) [35]. This discrepancy underscores that the cancer risk associated with tacrolimus may vary substantially across different patient populations and underlying diseases. In contrast to the findings in SLE and transplant recipients, a study of rheumatoid arthritis patients linked tacrolimus or methotrexate use, along with older age, to a significantly higher lymphoma risk (SIR = 3.43) [36]. Another study indicated that age was a predictor of extrathymic cancers in MG patients [37], and in our research, malignancies occurred in patients aged > 60 years after approximately three years of tacrolimus exposure. Although an association was observed, the observational nature of these data, including our own, precludes causal inference, and the influence of unmeasured confounders should be considered. Despite these concerns, the potential cancer risk associated with TAMO must be carefully weighed against its therapeutic benefits. The decision to initiate or continue TAMO should involve a shared decision‐making process. For patients aged ≤ 70 years or those with new‐onset MG, the likelihood of achieving MSE was higher, suggesting that the benefit–risk ratio may be most favorable in these populations. Conversely, for patients over 60 years of age, a more cautious assessment of the risk–benefit profile and rigorous cancer surveillance are warranted during long‐term tacrolimus therapy. Nevertheless, due to the small number of events and the retrospective nature of the data, this finding should be regarded as hypothesis‐generating and requires confirmation in larger, prospective cohorts. However, given the moderate sample sizes for the remission and relapse analyses and the particularly limited number of cancer events, these findings—especially the elevated SIR—should be interpreted as preliminary signals rather than definitive estimates, warranting validation in larger cohorts.

## 5 Limitation

This study has several limitations. Its retrospective design, single‐center design, and the absence of a control group may introduce selection and confounding biases, potentially affecting validity and generalizability.

First, the exclusion of other immunotherapies prior to tacrolimus initiation likely selected a study population enriched for milder or earlier‐stage disease. This potential selection bias may limit the generalizability of our findings to the broader MG population, which often requires more aggressive initial therapy. These findings, consistent with our previous report [5], suggest that TAMO could be appropriate for mild‐to‐moderate or new‐onset MG; however, its use in severe cases would need to be validated in future studies.

Second, our study enrolled an older population with a lower proportion of early‐onset MG (EOMG). This selection bias may limit the generalizability of our findings to younger patients, as the disease characteristics and treatment responses can vary between EOMG and late‐onset MG (LOMG).

Third, the taper speed was calculated as a single average value (total dose change over total time), which may oversimplify the typically non‐linear tapering process in clinical practice. Future prospective studies with protocol‐defined assessment intervals are needed to address this limitation.

Fourth, the moderate sample sizes for analyses (remission *n* = 153, relapse *n* = 100) may limit statistical power, and the small number of events in certain subgroups leads to imprecise estimates. This is particularly relevant for the interpretation of the elevated SIR for cancer, which was based on only eight events. Although the SIR reached statistical significance, the wide confidence interval indicates substantial uncertainty. Moreover, the observational nature of the study precludes causal inference, and the retrospective assessment of outcomes carries a potential for subjectivity. Therefore, the cancer risk finding should be regarded as a preliminary signal rather than a definitive estimate.

Fifth, the median follow‐up duration of 22.3 months is relatively short for robustly assessing long‐term relapse patterns and the highlighted cancer risk. The median time to cancer diagnosis in this study was 35.5 months, which exceeds the overall median follow‐up, suggesting that longer observation is required to fully capture potential malignancies.

Sixth, the absence of a control group is a key limitation, as it limits our ability to draw definitive conclusions about comparative efficacy or safety. Our analysis is therefore confined to absolute outcomes—such as time to MSE and relapse rates—which cannot be directly compared with other treatment modalities. While previous propensity‐matched analyses from our center have suggested comparable efficacy between tacrolimus and glucocorticoids in mild‐to‐moderate MG^5^, the applicability of these findings to broader or more severe patient populations remains uncertain. Thus, the efficacy, safety, and long‐term cancer risk of TAMO warrant confirmation in prospective, randomized controlled trials.

## 6 Conclusion

In conclusion, our study suggests that TAMO was associated with the achievement of MSE in adult‐onset mild‐to‐moderate MG patients, particularly in new‐onset cases and those with onset age ≤ 70 years. Furthermore, a tacrolimus concentration > 5.30 ng/mL at MSE and a dose reduction speed ≤ 1.08 mg/year were associated with a reduced risk of relapse. However, due to the limited follow‐up duration in this study, longer‐term studies are needed to confirm the long‐term safety of TAMO, particularly regarding cancer risk. Given the potential cancer risk, cancer surveillance may be considered for elderly patients on long‐term tacrolimus therapy.

## Funding

This work was supported by National Natural Science Foundation of China (62171299), Clinical Cohort Study of Myasthenia Gravis, National Key R&D Program of China, Precision Medicine Project (2017YFC0907700).

## Disclosure

The authors have nothing to report.

## Ethics Statement

This study was approved by the Ethics Committee of Xuanwu Hospital (No. 2017084), and each participant provided written informed consent.

## Conflicts of Interest

The authors declare no conflicts of interest.

## Supporting information


**Table S1:** Baseline characteristics of 153 patients included in remission analysis.
**Table S2:** Clinical characteristics of 200 patients enrolled in safety analysis.
**Figure S1:** Cumulative probability of MSE in all patients The estimated median period to MSE was 6.0 months (95% CI = 5.3–6.7). MSE, minimal symptom expression.
**Figure S2:** Kaplan–Meier survival curves used to determine the age‐at‐onset cut‐off value for predictor of MSE achievement. A cut‐off value of 70 years was identified. MSE, minimal symptom expression.
**Figure S3:** Kaplan–Meier‐derived cut‐off values for independent predictors of relapse.
(A) TAC concentration MSE: A cut‐off value of 5.30 ng/mL predicted relapse. (B) TAC dose reduction speed: A cut‐off value of 1.08 mg/year predicted relapse. TAC, tacrolimus; MSE, minimal symptom expression.

## Data Availability

The datasets used and/or analyzed during the current study are available from the corresponding author on reasonable request.

## References

[1] N. E. Gilhus , “Myasthenia Gravis,” New England Journal of Medicine 375, no. 26 (2016): 2570–2581. 28029925 10.1056/NEJMra1602678

[2] L. Yang , Y. Tang , F. He , et al., “Clinical Characteristics and Outcome Predictors of a Chinese Childhood‐Onset Myasthenia Gravis Cohort,” Frontiers in Pediatrics 10 (2022): 996213. 36245736 10.3389/fped.2022.996213 PMC9557758

[3] F. Pasqualin , S. V. Guidoni , M. Ermani , E. Pegoraro , and D. M. Bonifati , “Outcome Measures and Treatment Effectiveness in Late Onset Myasthenia Gravis,” Neurological Research and Practice 2 (2020): 45. 33324944 10.1186/s42466-020-00091-z PMC7650071

[4] Z. Fan , Z. Li , F. Shen , et al., “Favorable Effects of Tacrolimus Monotherapy on Myasthenia Gravis Patients,” Frontiers in Neurology 11 (2020): 594152. 33193063 10.3389/fneur.2020.594152 PMC7652845

[5] Z. Fan , L. Lei , S. Su , et al., “Comparison Between Mono‐Tacrolimus and Mono‐Glucocorticoid in the Treatment of Myasthenia Gravis,” Annals of Clinical and Translational Neurology 10, no. 4 (2023): 589–598. 36808840 10.1002/acn3.51746 PMC10109324

[6] D. P. Richman and M. A. Agius , “Treatment of Autoimmune Myasthenia Gravis,” Neurology 61, no. 12 (2003): 1652–1661. 14694025 10.1212/01.wnl.0000098887.24618.a0

[7] S. C. Ong and R. S. Gaston , “Thirty Years of Tacrolimus in Clinical Practice,” Transplantation 105, no. 3 (2021): 484–495. 32541562 10.1097/TP.0000000000003350

[8] N. E. Gilhus and J. J. Verschuuren , “Myasthenia Gravis: Subgroup Classification and Therapeutic Strategies,” Lancet Neurology 14, no. 10 (2015): 1023–1036. 26376969 10.1016/S1474-4422(15)00145-3

[9] J. Palace , J. Newsom‐Davis , and B. Lecky , “A Randomized Double‐Blind Trial of Prednisolone Alone or With Azathioprine in Myasthenia Gravis. Myasthenia Gravis Study Group,” Neurology 50, no. 6 (1998): 1778–1783. 9633727 10.1212/wnl.50.6.1778

[10] D. B. Sanders , I. K. Hart , R. Mantegazza , et al., “An International, Phase III, Randomized Trial of Mycophenolate Mofetil in Myasthenia Gravis,” Neurology 71, no. 6 (2008): 400–406. 18434638 10.1212/01.wnl.0000312374.95186.cc

[11] J. Morren and Y. Li , “Maintenance Immunosuppression in Myasthenia Gravis, an Update,” Journal of the Neurological Sciences 410 (2020): 116648. 31901719 10.1016/j.jns.2019.116648

[12] K. Itani , M. Nakamura , R. Wate , et al., “Efficacy and Safety of Tacrolimus as Long‐Term Monotherapy for Myasthenia Gravis,” Neuromuscular Disorders 31, no. 6 (2021): 512–518. 33903022 10.1016/j.nmd.2021.02.010

[13] W. Duan , Y. Peng , W. Jin , S. Ouyang , and H. Yang , “Tacrolimus as Single‐Agent Immunotherapy and Minimal Manifestation Status in Nonthymoma Myasthenia Gravis,” Journal of Immunology Research 2021 (2021): 9138548. 34845439 10.1155/2021/9138548 PMC8627335

[14] Z. Bi , Y. Cao , C. Liu , et al., “Remission and Relapses of Myasthenia Gravis on Long‐Term Tacrolimus: A Retrospective Cross‐Sectional Study of a Chinese Cohort,” Therapeutic Advances in Chronic Disease 13 (2022): 20406223221122538. 36093262 10.1177/20406223221122538 PMC9459458

[15] Y. Nishida , Y. K. Takahashi , T. Kanai , et al., “Safety of Tapering Tacrolimus Dose in Patients With Well‐Controlled Anti‐Acetylcholine Receptor Antibody‐Positive Myasthenia Gravis,” European Journal of Neurology 27, no. 1 (2020): 100–104. 31309642 10.1111/ene.14039

[16] J. Vissing , S. Jacob , K. P. Fujita , F. O'Brien , and J. F. Howard , “'Minimal Symptom Expression' in Patients With Acetylcholine Receptor Antibody‐Positive Refractory Generalized Myasthenia Gravis Treated With Eculizumab,” Journal of Neurology 267, no. 7 (2020): 1991–2001. 32189108 10.1007/s00415-020-09770-y PMC7320935

[17] Neuroimmunology Branch of the Chinese Society of I , “Chinese Guidelines for Diagnosis and Treatment of Myasthenia Gravis (2025 Edition),” Chinese Journal of Neuroimmunology and Neurology 58, no. 7 (2025): 721–741.

[18] T. Takeuchi , N. Wakasugi , T. Hashida , S. Uno , and H. Makino , “Long‐Term Safety and Effectiveness of Tacrolimus in Patients With Lupus Nephritis in Japan: 10‐Year Analysis of the Real‐World TRUST Study,” Journal of Rheumatology 51, no. 6 (2024): 613–621. 38359944 10.3899/jrheum.2023-0210

[19] F. Piehl , A. Eriksson‐Dufva , A. Budzianowska , et al., “Efficacy and Safety of Rituximab for New‐Onset Generalized Myasthenia Gravis: The RINOMAX Randomized Clinical Trial,” JAMA Neurology 79, no. 11 (2022): 1105–1112. 36121672 10.1001/jamaneurol.2022.2887 PMC9486640

[20] D. B. Sanders , G. I. Wolfe , M. Benatar , et al., “International Consensus Guidance for Management of Myasthenia Gravis: Executive Summary,” Neurology 87, no. 4 (2016): 419–425. 27358333 10.1212/WNL.0000000000002790 PMC4977114

[21] T. Kanai , A. Uzawa , N. Kawaguchi , et al., “Adequate Tacrolimus Concentration for Myasthenia Gravis Treatment,” European Journal of Neurology 24, no. 2 (2017): 270–275. 28102047 10.1111/ene.13189

[22] R. S. Zheng , R. Chen , B. F. Han , et al., “Cancer Incidence and Mortality in China, 2022,” Zhonghua Zhong Liu Za Zhi 46, no. 3 (2024): 221–231. 38468501 10.3760/cma.j.cn112152-20240119-00035

[23] H. Murai , K. Utsugisawa , Y. Nagane , S. Suzuki , T. Imai , and M. Motomura , “Rationale for the Clinical Guidelines for Myasthenia Gravis in Japan,” Annals of the New York Academy of Sciences 1413, no. 1 (2018): 35–40. 29377151 10.1111/nyas.13544

[24] Y. Nagane , S. Suzuki , N. Suzuki , and K. Utsugisawa , “Factors Associated With Response to Calcineurin Inhibitors in Myasthenia Gravis,” Muscle & Nerve 41, no. 2 (2010): 212–218. 19816912 10.1002/mus.21462

[25] N. Xie , Q. Liu , Q. Wen , et al., “Short‐Term and Long‐Term Prognoses in AChR‐Ab Positive Very‐Late‐Onset Myasthenia Gravis Patients,” Therapeutic Advances in Neurological Disorders 18 (2025): 17562864241309793. 39803329 10.1177/17562864241309793 PMC11713957

[26] Y. Zheng , X. Yuan , C. Zhang , et al., “Efficacy and Safety of Tacrolimus Therapy for a Single Chinese Cohort With Very‐Late‐Onset Myasthenia Gravis,” Frontiers in Neurology 13 (2022): 843523. 35432159 10.3389/fneur.2022.843523 PMC9007732

[27] M. C. Dalakas and J. Yi , “Late‐Onset Stiff‐Person Syndrome: Challenges in Diagnosis and Management,” Therapeutic Advances in Neurological Disorders 16 (2023): 17562864231214315. 38152088 10.1177/17562864231214315 PMC10752047

[28] C. Zhang , B. Bu , H. Yang , et al., “Immunotherapy Choice and Maintenance for Generalized Myasthenia Gravis in China,” CNS Neuroscience & Therapeutics 26, no. 12 (2020): 1241–1254. 33103369 10.1111/cns.13468 PMC7702233

[29] J. Verwijst , E. Westerberg , and A. R. Punga , “Cancer in Myasthenia Gravis Subtypes in Relation to Immunosuppressive Treatment and Acetylcholine Receptor Antibodies: A Swedish Nationwide Register Study,” European Journal of Neurology 28, no. 5 (2021): 1706–1715. 33427389 10.1111/ene.14730

[30] Y. Yagi , N. Sanjo , T. Yokota , and H. Mizusawa , “Tacrolimus Monotherapy: A Promising Option for Ocular Myasthenia Gravis,” European Neurology 69, no. 6 (2013): 344–345. 23549260 10.1159/000347068

[31] X. Tao , W. Wang , F. Jing , et al., “Long‐Term Efficacy and Side Effects of Low‐Dose Tacrolimus for the Treatment of Myasthenia Gravis,” Neurological Sciences: Official Journal of the Italian Neurological Society and of the Italian Society of Clinical Neurophysiology 38, no. 2 (2017): 325–330. 27873026 10.1007/s10072-016-2769-5

[32] I. Rodríguez‐Lago , J. Castro‐Poceiro , A. Fernández‐Clotet , et al., “Tacrolimus Induces Short‐Term but Not Long‐Term Clinical Response in Inflammatory Bowel Disease,” Alimentary Pharmacology & Therapeutics 51, no. 9 (2020): 870–879. 32181930 10.1111/apt.15687

[33] M. Rodríguez‐Perálvarez , J. Colmenero , A. González , et al., “Cumulative Exposure to Tacrolimus and Incidence of Cancer After Liver Transplantation,” American Journal of Transplantation 22, no. 6 (2022): 1671–1682. 35286761 10.1111/ajt.17021 PMC9315045

[34] K. Ichinose , S. Sato , T. Igawa , et al., “Evaluating the Safety Profile of Calcineurin Inhibitors: Cancer Risk in Patients With Systemic Lupus Erythematosus From the LUNA Registry‐a Historical Cohort Study,” Arthritis Research & Therapy 26, no. 1 (2024): 48. 38347556 10.1186/s13075-024-03285-x PMC10860233

[35] P. Maisonneuve , A. B. Lowenfels , and B. C. Marshall , “Risk of Colorectal Cancer After Solid Organ Transplantation in the United States,” American Journal of Transplantation 16, no. 8 (2016): 2498. 27104677 10.1111/ajt.13833

[36] A. Hashimoto , N. Chiba , H. Tsuno , et al., “Incidence of Malignancy and the Risk of Lymphoma in Japanese Patients With Rheumatoid Arthritis Compared to the General Population,” Journal of Rheumatology 42, no. 4 (2015): 564–571. 25593236 10.3899/jrheum.140533

[37] C. J. Liu , Y. S. Chang , C. J. Teng , et al., “Risk of Extrathymic Cancer in Patients With Myasthenia Gravis in Taiwan: A Nationwide Population‐Based Study,” European Journal of Neurology 19, no. 5 (2012): 746–751. 22221515 10.1111/j.1468-1331.2011.03621.x

