# Supplementary material for: Tacrolimus as Single‐Agent Immunotherapy for Adult‐Onset Myasthenia Gravis: Remission, Relapse, and Safety
Source: CNS Neurosci Ther. 2026 Jun 1;32(6):e70921. doi: 10.1002/cns.70921 (PMC13238655; doi:10.1002/cns.70921)
Supplement: Supplementary file 1 — Table S1: Baseline characteristics of 153 patients included in remission analysis. Table S2: Clinical characteristics of 200 patients enrolled in safety analysis. Figure S1: Cumulative probability of MSE in all patients The estimated median period to MSE was 6.0 months (95% CI = 5.3–6.7). MSE, minimal symptom expression. Figure S2: Kaplan–Meier survival curves used to determine the age‐at‐onset cut‐off value for predictor of MSE achievement. A cut‐off value of 70 years was identified. MSE, minimal symptom expression. Figure S3: Kaplan–Meier‐derived cut‐off values for independent predictors of relapse. (A) TAC concentration MSE: A cut‐off value of 5.30 ng/mL predicted relapse. (B) TAC dose reduction speed: A cut‐off value of 1.08 mg/year predicted relapse. TAC, tacrolimus; MSE, minimal symptom expression. [file CNS-32-e70921-s001.docx]

**Table S1.** Baseline characteristics of 153 patients included in remission analysis

| **Characteristics** |  |
| --- | --- |
| Sex, n (%) |  |
| Female | 85 (55.6) |
| Male | 68 (44.4) |
| Age at TAC initiation, years (IQR) | 62.0 (50.0-69.0) |
| Age at onset, years (IQR) | 61.0 (49.5-68.0) |
| 18-49, n (%) | 38 (24.8) |
| 50-64, n (%) | 56 (36.6) |
| ≥65, n (%) | 59 (38.6) |
| MGFA classification at baseline, n (%) |  |
| I | 34 (22.2) |
| II | 89 (58.2) |
| III | 30 (19.6) |
| MG-ADL score at baseline, (IQR) | 5 (3-6) |
| QMG score at baseline, (IQR) | 7 (5-11) |
| Symptoms at baseline, n (%) |  |
| Ocular | 144 (94.1) |
| Bulbar | 61 (39.8) |
| Neck or limb | 110 (71.9) |
| Respiratory | 16 (10.4) |
| Thymoma, n (%) | 14 (9.8) |
| Thymectomy, n (%) | 15 (9.9) |
| RNS, n (%) |  |
| Positive | 71/114 (62.3) |
| Negative | 43/114 (37.7) |
| AChR-Ab (+), n (%) | 131/149 (87.9) |
| Time to diagnosis, months (IQR) | 2.0 (0.8-9.1) |
| Disease duration, months (IQR) | 7.0 (2.3-21.8) |
| New-onset MG, n (%) | 92 (60.1) |
| Comorbidities, n (%) |  |
| Hypertension | 63 (41.2) |
| Diabetes | 30 (19.6) |
| Coronary heart disease | 18 (11.8) |
| Thyroid disease | 14 (9.2) |
| Autoimmune disease | 6 (3.9) |
| Follow-up time, month (IQR) | 24.4 (10.6-43.4) |
| MG, myasthenia gravis; TAC, tacrolimus; IQR, interquartile range; MGFA classification, Myasthenia Gravis Foundation of America classification; MG-ADL, Myasthenia Gravis Activities of Daily Living; QMG, quantitative myasthenia gravis; RNS, repetitive nerve stimulation; AChR-Ab, acetylcholine receptor antibodies. | |

**Table S2.** Clinical characteristics of 200 patients enrolled in safety analysis

| **Characteristics** |  |
| --- | --- |
| Sex, n (%) |  |
| Female | 100 (50.0) |
| Male | 100 (50.0) |
| Age at TAC initiation, years (IQR) | 61.0 (46.25-68.0) |
| Disease duration, months (IQR) | 7.1(2.5-23.0) |
| Comorbidities, n (%) |  |
| Hypertension | 81 (40.5) |
| Diabetes Mellitus | 39 (19.5) |
| Coronary atherosclerotic heart disease | 21 (10.5) |
| Thymoma, n (%) | 24/189 (12.7) |
| AChR-Ab (+), n (%) | 164/186 (88.2) |
| RNS (+), n (%) | 91/141 (64.5) |
| Follow-up time, months (IQR) | 22.3 (7.5-40.5) |
| TAC, tacrolimus; IQR, interquartile range; AChR-Ab, acetylcholine receptor antibodies; RNS, repetitive nerve stimulation. | |


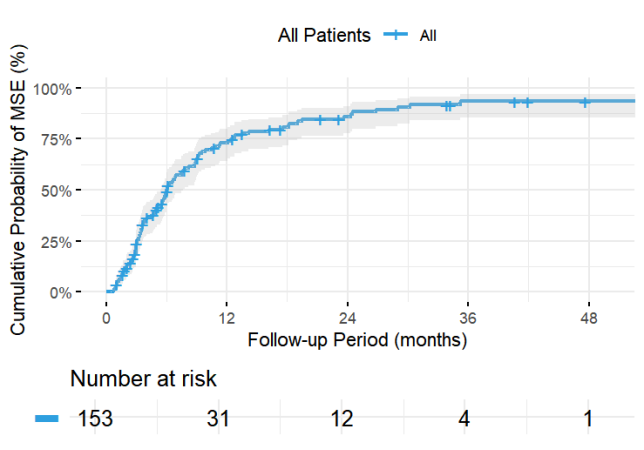


**Figure S1.** Cumulative probability of MSE in all patients.The estimated median period to MSE was 6.0 months (95% CI = 5.3–6.7). MSE, minimal symptom expression.


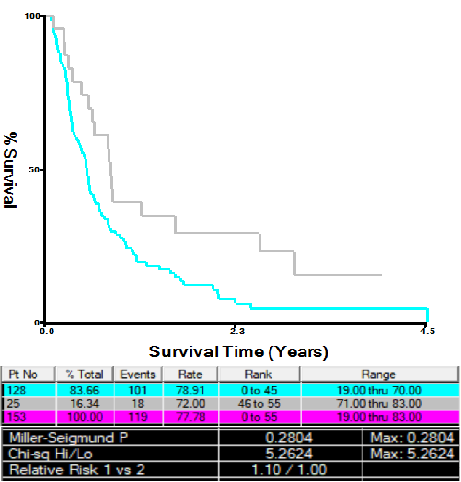


**Figure S2.** Kaplan–Meier survival curves used to determine the age-at-onset cut-off value for predictor of MSE achievement. A cut-off value of 70 years was identified. MSE, minimal symptom expression.


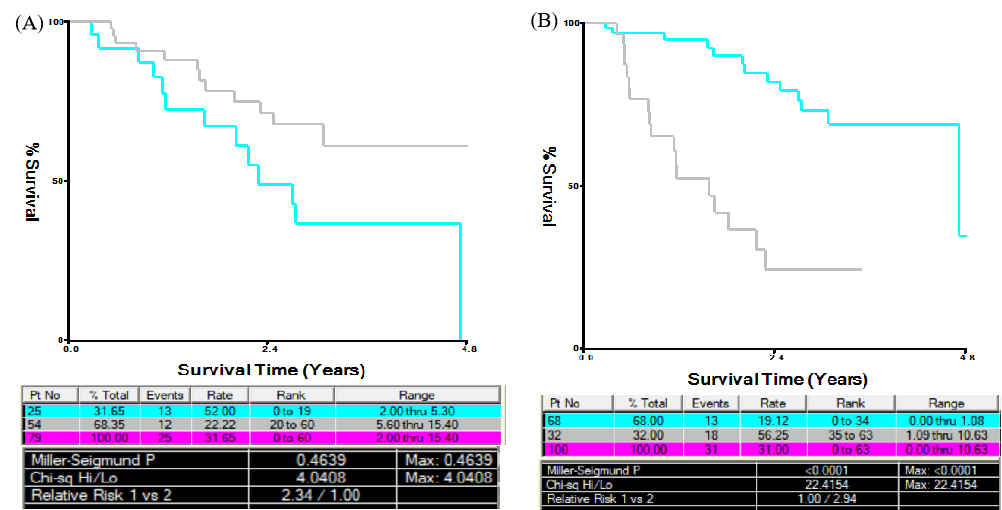


**Figure S3.** Kaplan-Meier-derived cut-off values for independent predictors of relapse.
(A) TAC concentration MSE: A cut-off value of 5.30 ng/mL predicted relapse. (B) TAC dose reduction speed: A cut-off value of 1.08 mg/year predicted relapse. TAC, tacrolimus; MSE, minimal symptom expression.
